# Is the Habitual Dietary Intake of Foods of Plant or Animal Origin Associated with Circulating Hemostatic Factors?—Results of the Population-Based KORA-Fit Study

**DOI:** 10.3390/nu16030432

**Published:** 2024-01-31

**Authors:** Michael Schepp, Dennis Freuer, Annette Peters, Margit Heier, Daniel Teupser, Christine Meisinger, Jakob Linseisen

**Affiliations:** 1 Epidemiology, University Hospital Augsburg, University of Augsburg, 86156 Augsburg, Germany; dennis.freuer@med.uni-augsburg.de (D.F.); christine.meisinger@med.uni-augsburg.de (C.M.); jakob.linseisen@med.uni-augsburg.de (J.L.); 2 Institute of Epidemiology, Helmholtz Zentrum München—German Research Center for Environmental Health (GmbH), 85764 Neuherberg, Germany; annette.peters@helmholtz-munich.de (A.P.); margit.heier@helmholtz-munich.de (M.H.); 3 Chair of Epidemiology, Institute for Medical Information Processing, Biometry and Epidemiology, Medical Faculty, Ludwig-Maximilians-Universität München, 81377 Munich, Germany; 4 German Center for Diabetes Research (DZD), 85764 Neuherberg, Germany; 5 KORA Study Centre, University Hospital Augsburg, 86156 Augsburg, Germany; 6 Institute of Laboratory Medicine, Medical Faculty, Ludwig-Maximilians-Universität München, 81377 Munich, Germany; daniel.teupser@med.uni-muenchen.de

**Keywords:** nutrition, diet, coagulation, hemostatic factors, food groups, KORA

## Abstract

Blood coagulation is a complex physiological process critical for maintaining hemostasis, and disruptions in this system can lead to various health complications. Since the effects of specific food groups on a series of circulating coagulation parameters in the population are not well established, this study examines such associations in the population-based KORA-Fit study. A total of 595 subjects (263 men and 332 women) born between 1945 and 1964 and living in the study region of Augsburg were included in the study. Habitual food intake was estimated based on a combination of repeated 24-h food lists (24HFLs) and a food frequency questionnaire (FFQ). Antithrombin III, D-dimers, factor VIII, fibrinogen, protein C, protein S, aPTT, Quick value and INR were measured in citrate plasma. Multivariable linear regression models were applied to investigate associations between the consumption of specific foods of plant or animal origin and hemostatic factors. We found that the consumption of plant-based food groups, including green leafy vegetables (rich in vitamin K1), were hardly associated with coagulation parameters. Surprisingly, a high consumption of dairy products and especially butter were associated with higher D-dimer concentrations. These findings need further evaluation in prospective studies.

## 1. Introduction

According to the WHO, non-communicable diseases are the main cause of deaths worldwide and are responsible for the death of around 41 million people per year [1]. Eighty percent of these cases are caused by chronic conditions like cancer, diabetes, respiratory and cardiovascular diseases (CVD) which are often affected by lifestyle factors such as smoking, physical inactivity or an unhealthy diet [2]. Especially the importance of a healthy eating behavior has emerged in the past years as an effective primary prevention strategy for several of the mentioned diseases. For example, a comprehensive systematic review and meta-analysis of 95 prospective studies has reported an inverse dose-response relationship between fruit and vegetable consumption and cardiovascular disease, total cancer and all-cause mortality [3]. Also, adherence to favorable dietary patterns, such as the Mediterranean Diet Score (MDS), which is characterized by a high intake of plant-based food items like fruit and vegetables, whole grains, and nuts but also a moderate number of animal-derived foods such as fish, has been shown to yield protective effects regarding CVD, cancer and the metabolic syndrome in several trials [4].

The reduction of CVD risk by following a Mediterranean-style diet is thought to be mainly mediated by its bioactive compounds which have antioxidant, anti-inflammatory and, interestingly, also anti-thrombotic properties [5,6]. There is also evidence that the risk for thrombosis is mediated by pro-inflammatory processes in the human body [7] but less is known about the specific effects on coagulation parameters which play a critical role in maintaining hemostasis and preventing excessive bleeding or coagulation (thrombosis), [8]. Therefore, the influence of habitual food consumption on these parameters is an area that warrants more investigation.

Indeed, positive effects of some specific nutrients like long-chain omega-3-fatty acids derived mainly from fish and seafood, such as eicosapentaenoic acid (EPA) and docosahexaenoic acid (DHA), on different hemostatic parameters have been observed in large cross-sectional studies [9,10] as well as in controlled intervention studies [11,12] that also may explain some of the beneficial effects of the MDS on thrombosis risk. On the other hand, the effect on coagulation parameters of other antioxidant-rich, plant-based diets such as a vegetarian diet pattern showed more mixed results [13,14] and it seems that the effects on hemostasis are more influenced by specific food choices rather than the specific dietary pattern [14]. While some studies examined the outcomes of different dietary patterns or single nutrients on coagulation parameters [14,15], to our knowledge, consumption of foods, differentiating between plant- and animal-derived food items, has not been investigated comprehensively yet at the population-based level.

Therefore, the present study aims to uncover associations between habitual food consumption with several coagulation factors.

## 2. Materials and Methods

### 2.1. Study Sample

The study sample of our project is part of the KORA S4 Fit survey. KORA (Cooperative Health Research in the Region of Augsburg) is the continuation of the WHO MONICA (Monitoring Trends and Determinants in Cardiovascular Disease) project which was conducted by the WHO from 1984 to 1995 [16]. In 1996, the KORA study was established by the Helmholtz Center Munich—National Research Center for Environment and Health and is a prospective population-based adult cohort study in the region of Augsburg, Germany [16].

Over the time course, a total of four cross-sectional baseline surveys from 1984 to 2001 (S1 to S4) were initiated with 18,000 participants being randomly selected and follow-up studies were conducted in intervals ranging from 4 to 20 years [17]. The KORA S4 survey included a sample of 4261 inhabitants of the study region (city of Augsburg and two surrounding counties) aged 25 to 74 years and was performed from 1999 to 2001. KORA-Fit is a follow-up study conducted from 2018 to 2019 with 3059 subjects from the S1–S4 cross-sectional surveys born between 1945 and 1964 [17].

The ethical release was provided by the Ethics Committee of the Bavarian Medical Association (Bayerische Landesärztekammer). All study participants gave written informed consent, and the study was conducted in accordance with the Declaration of Helsinki [16].

Our study sample originally consisted of 624 subjects of the S4 part of KORA-Fit who had available food intake data and blood plasma measurements of hemostatic parameters. After the exclusion of participants that were currently receiving anticoagulative treatment (n = 29), a total of 595 subjects (263 men and 332 women) remained for the analysis.

### 2.2. Measurements of Exposures

For anthropometric measurements, participants were encouraged to remove heavy clothing and shoes to determine their body weight and height, according to the WHO MONICA protocol [18]. Waist circumference was measured between the distance of the lower rib margin and the iliac crest [19]. The body mass index (BMI) of the subjects was calculated by dividing the weight (in kg) by the square of the height (in m). Other exposure variables such as smoking (current/former/never), education years (more or less than 12 years), physical activity (estimated time per week spent on sports activities during leisure time in summer and winter), and medication use were assessed during a standardized face-to-face interview by instructed medical personnel. Subjects were defined as having diabetes if they got a medical diagnosis and/or were under antidiabetic medication. Similarly, to be classified in the hypertension category, participants had a baseline blood pressure of systolic ≥ 140 or diastolic ≥ 90 mmHg or received an antihypertensive medical treatment given previously diagnosed with hypertension [18].

The usual dietary intake was assessed using repeated 24-h food lists (24HFLs) and a food frequency questionnaire (FFQ) asking about the habitual diet over the past 12 months which is based on the self-administered German version of the European Food Propensity Questionnaire (EFPQ) [20,21]. By combining both data sets as described previously by Mitry et al. [20] the habitual consumption of food items was estimated in g/day. Food items were summarized into food groups and subgroups; the analysis focused on foods of plant and animal origin including total fruits, total vegetables, green leafy vegetables (as a subgroup of vegetables), total meat, total fish, total eggs, and dairy products (w/o butter), cheese (as a subgroup of dairy products) and butter. As confounding variables, we used the estimated intake of alcohol (in grams per day) and total energy (in kilocalories per day).

In our study we assessed selected hemostatic parameters that exert different functions in the pathways of the coagulation cascade. Factor VIII is part of the intrinsic pathway whereas in the common pathway, fibrinogen is converted into fibrin strands by thrombin (factor II) which then finally get cross-linked, catalyzed by factor XIII [8]. The activation of the intrinsic pathway can be overall quantified by the aPTT which gets lowered in a pro-coagulant state while the quick value measures how the extrinsic pathway is affected and is inversely correlated with clotting tendency [22]. In the scientific literature, often the prothrombin time is used instead of the quick value to analyze the extrinsic pathway. Also, the INR value is another method to determine the activation of the extrinsic pathway in a more standardized manner and gets elevated in a pro-thrombogenic environment [22]. Antithrombin III is anti-thrombogenic molecule that works as a protease inhibitor and mainly exhibits its effect by the inactivation of thrombin. Proteins C and S are vitamin K-dependent factors that also work as inhibitors of the coagulation cascade. Both are synthesized by the liver where also most of the other factors of the coagulation system were produced [22]. D-dimers are degradation products of cross-linked fibrin and are widely recognized as a biomarker for assessing the activation of the coagulation system [23,24].

Measurements of standard clinical parameters were conducted in the central laboratory of Ludwig-Maximilians-University Munich, while hemostatic variables were measured in citrate plasma in the central lab of the University Hospital Augsburg by instructed laboratory staff, and reference values were derived from the laboratory reference sheets [25].

Non-HDL-cholesterol (non-HDLc) was defined as the difference between total cholesterol and high-density lipoprotein cholesterol (HDLc). Fatty liver index (FLI) was calculated by using the FLI formula previously described by Bedogni et al. which includes laboratory measurements of triglycerides (TG) and gamma-glutamyl transferase (GGT) [26].

The variables and methods regarding the laboratory measurements are described in Table 1 [25,27].

### 2.3. Statistical Analyses

Statistical analyses were performed by using the R-software (R version 4.3.1). The Shapiro–Wilk test was used to test for normal distribution. Because the continuous parameters were not normally distributed, these variables are described by its median and 25th–75th percentile range. All categorial variables were described as absolute or relative frequencies. To analyze sex differences for continuous variables the Mann–Whitney U test was applied, while for categorial variables Fisher’s exact test was utilized. A *p*-value < 0.05 was defined as statistically significant.

In the linear regression models the coagulation parameters were used as the dependent variables and food consumption data as independent variables. The ß-estimate describes the effect size of each parameter by displaying the estimated change of the dependent variable when the independent variable changes by one unit. A positive or negative sign of the ß-estimate indicates the direction of the association. To control for possible confounding variables, each model was adjusted for sex, age, physical activity, education years, smoking, diabetes, hypertension, total energy intake, alcohol consumption, non-HDLc, and BMI. By using the Variance-Inflation-Factor (VIF) we observed significant multicollinearity for two of the adjustment variables, BMI and FLI; thus, in sensitivity analyses, BMI was replaced by FLI. To secure all necessary model assumptions we tested for autocorrelation, heteroscedasticity, normality, linearity and removed extreme outliers to create more precise models. We tested for interaction effects by sex. We took into account multiple testing by using the Benjamini–Hochberg False Discovery Rate (FDR) method and calculated adjusted *p*-values. Finally, the significant models were additionally adjusted for hs-CRP to investigate if inflammatory conditions may have contributed to the observed effects.

## 3. Results

Table 2 shows the characteristics of the male and female participants. There were significant sex differences for BMI, waist circumference, total energy intake, alcohol consumption, FLI, CRP, education years, hypertension and diabetes regarding men (versus women) showing higher values in all mentioned variables, except for education years.

Table 3 sums up the plasma concentrations of blood coagulation factors of the study participants. For male participants, significantly lower median values for antithrombin III, protein C and Quick value levels were observed as compared to women. On the opposite, in women significantly higher median levels for protein S and INR were noted. All median values were within the laboratory reference ranges. Furthermore, there was a large proportion of participants (n = 190) with D-dimer values above the reference range (≥500 µg/L). The exact numbers of participants with measurements outside the reference range of coagulation factors can be found in the Supplementary Materials (Table S1).

For men, a higher consumption of foods of animal origin, including meat, fish and butter was observed, whereas for women a higher consumption of fruits, vegetables and dairy products was found (Table 4).

Table 5, Table 6 and Table 7 show the associations between the food group variables as independent factors and the blood coagulation parameters as dependent variables. Statistically significant positive associations for total fruit intake and aPTT (*p* = 0.036), dairy product intake and D-dimers (*p* = 0.032), butter intake and D-dimers (*p* < 0.001) as well as butter intake and protein C (*p* = 0.047) were observed. Inverse associations were shown for dairy products and antithrombin III (*p* = 0.010) and protein C (*p* = 0.001), respectively.

After correction for multiple testing only the associations between butter consumption and D-dimers (*p* < 0.001) and dairy product intake and protein C (*p* = 0.040) remained statistically significant. To identify a possible effect of inflammation on D-dimers or protein C, we additionally adjusted for hs-CRP in these two models. Still, statistical significance was observed for the butter and D-dimer model (*p* = 0.016) as well as for the dairy product and protein C model (*p* = 0.017). With the adjustment for FLI replacing BMI similar results were observed; however, after FDR correction, none of the associations remained statistically significant (Supplementary Materials, Tables S2–S4).

Additionally, because of a large proportion of participants with elevated D-dimer values [>500 µg/L], we stratified the study subjects into two subgroups depending on their D-dimer levels and created two new linear regression models investigating the association between D-dimers and butter intake [g/d]. After transforming the ß-estimates, for the subgroup with D-dimers within the reference value (n = 405) we observed a ß-estimate of 1.634 (95% CI 0.992; 2.694) with a *p*-value of 0.054. For the subgroup with elevated D-dimers (n = 190) the transformed ß-estimate was 0.842 (95% CI 0.246; 2.877) and the *p*-value was 0.783.

## 4. Discussion

### 4.1. Main Findings

As main findings we observed that a higher butter intake is associated with significantly higher values for D-dimers; this association with D-dimers is also nominally significant (i.e., before correction for multiple correction) for the consumption of dairy products. Elevated D-dimer levels are typically associated with conditions such as thrombosis, disseminated intravascular coagulation or inflammation [23,24]. Interestingly, D-dimers also seem to increase with ageing and elevated values seem to be more common in middle-aged or older individuals [30]. The findings on protein C are conflicting, i.e., a significantly inverse association with the consumption of dairy products, and a (nominally) positive association with butter consumption. Among the investigated plant-derived foods, only the intake of fruits was significantly associated with higher aPTT values, and this association disappeared after FDR correction.

### 4.2. Butter, Dairy Products and Cheese

Besides the increasing effect on D-dimers, butter consumption as well seems to exert inhibitory effects on hemostasis by the association with higher protein C values while no effect for other coagulation parameters, especially for the functional tests aPTT, INR or Quick value were observed. In addition, a randomized cross-over study observed no direct effects of butter consumption on several coagulation parameters [31]. Saturated fatty acids (SFA) of different chain lengths are main nutritional components of butter [32]. And, regarding hemostasis, some pro-coagulant parameters like factor VII were previously shown to be elevated when (long-chain) SFAs were consumed instead of unsaturated fatty acids (UFA) while for other factors like antithrombin III or fibrinogen no effect was observed [33]. Another randomized cross-over study by Delgado-Lista et al. compared the effects of a diet rich in SFA mainly derived from butter with diets rich in UFA or refined carbohydrates on fasting and post-prandial hemostatic factors after 28-days intervention periods for each diet [34]. After the intervention there were no significant changes shown in measured parameters such as factor VII and D-dimers in a fasted state. However, 4 h after consuming a fat-enriched meal, for factor VII significantly lower values in the UFA diet group were observed whereas D-dimers were significantly elevated in all of the three groups with the largest increase after SFA consumption [34]. While similar effects of dietary fatty acids on fasting and postprandial factor VII measurements were also reported in previous studies as summarized in a review by Pieters et al. [15], the mechanism of these observations is still not fully established yet as well as effects of specific food items on D-dimers which were observed in our study for butter and dairy intake. Besides coagulation system activation, D-dimers are also recognized as a marker for inflammatory processes [23] which could indicate a non-favorable effect of a diet rich in SFA derived from these food items. However, even after the adjustment for CRP, the association between butter and D-dimers remained significant which indicates that other mechanisms apart from inflammation could also be a reason for this observation. While the observed association seems to be more pronounced in individuals which are still within the reference range of D dimers (versus participants above the reference range), none of the additional models were able to reach statistical significance.

On the other hand, regarding CVD risk and direct pro-coagulant effects, dairy intake generally seems to yield neutral or protective effects [35]. Besides its saturated fat content, milk is also a great source of nutrients that have been shown to be inversely associated with CVD risk like vitamin K2 [36], milk protein [37] or contain other bioactive compounds such as specific biopeptides that exhibit anti-thrombotic and antioxidant activities [38,39]. Vitamin K2, especially, which is mainly derived from ruminant meat and dairy products [40], is also directly involved in the coagulation process by the carboxylation of several hemostatic factors such as factor II, VII, IX and X [8].

It was surprising that we observed an association of dairy products consumption with higher D-dimers and lower values for protein C and antithrombin III which indicates an overall pro-coagulant effect. However, especially for cheese which is besides butter one of the dairy products richest in saturated fatty acids (per g food), we could not report similar findings. Also, in a meta-analysis of prospective studies an inverse relationship for CVD risk was observed for cheese consumption [41]. Possible explanations could be the role of beneficial nutrients that are especially found in higher concentrations in fattier dairy products such as conjugated linoleic acid which has been shown to beneficially influence inflammatory parameters and platelet aggregation markers in a cross-over intervention study [42]. Cheese also contains significant amounts of probiotics like Lactobacillus casei which has been found to reduce pro-inflammatory and pro-coagulant factors in an experimental model [43]. To our knowledge no trials on humans exist to date that comprehensively analyzed the direct effects of dairy product consumption on hemostatic factors.

Despite being linked to inflammatory processes [44], the associations of protein C with dairy products still remained significant after correction for multiple testing as well as after the additional adjustment for CRP. This observation, together with the findings on butter intake and associated increment of D-dimer values, may indicate possible unfavorable effects of these food items on thrombosis risk.

### 4.3. Fish, Eggs and Meat

In contrast to dairy products, for total fish, total egg, and total meat consumption no significant associations with any blood coagulation parameter were observed. Especially for fish intake which goes along with a high intake of long-chain omega-3-fatty acids, an antithrombotic effect could be expected based on the results of interventional studies which showed a positive effect on aPTT [45] as well as a reduction of prothrombin time [46] which could also be applied for the Quick value. In epidemiological studies like ARIC (Atherosclerosis Risk In Communities) and CARDIA (Coronary Artery Risk Development In young Adults) more mixed findings for fish intake were reported [9,47]. In the ARIC study fish intake was around 20 g per day (1.4 servings of 3–5 ounces of fish per week) while on average 10.8 g fish per day were consumed in the CARDIA study. While no significant results were reported in the CARDIA study, the ARIC study found significant inverse associations for fibrinogen, factor VIII and Von Willebrand factor which could indicate an anticoagulant effect of an increased fish intake. However, such a high fish intake is rarely seen in the general population, and this could also be an explanation for the no significant results in the CARDIA study. In our study the mean fish intake was around 18.2 g per day which could also possibly be too low to observe any effects.

For egg consumption, to date only few studies examined the effects on blood coagulation parameters. In a prospective study by Vorster et al. no effects on coagulation factors were found when egg intake was increased from three eggs per week for 2 months to 7 or 14 eggs per week for 5 months [48]. In the ATTICA study, a large prospective observational study with 3042 subjects, especially for low SFA intakes, an inverse correlation with fibrinogen was shown for an increased egg intake suggesting an anticoagulant effect of eggs [49]. Apart from their high cholesterol content, eggs contain a variety of essential nutrients such as zinc, iodine, B-vitamins and also bioactive compounds like phospholipids or the carotenoids lutein and zeaxanthin that yield anti-oxidative and anti-inflammatory effects [50,51]. Eggs generally seem to be safe to be consumed on a regular basis and do not appear to significantly affect blood coagulation parameters as observed in our study [52].

Regarding meat intake, there is also only weak evidence for an association with hemostatic factors. The complete restriction of meat intake was analyzed in studies with vegetarians and yielded more inconclusive findings [13,14] while only few studies investigated the direct effects of increased meat intake on coagulation parameters. In a cross-sectional study from 1991 in 995 Japanese subjects, no association between total meat consumption with fibrinogen was found [53]. Also, in a randomized controlled trial which examined the effect of red meat intake on fibrinogen levels, no significant effect was observed [54]. Meat also contains bioactive compounds such as L-carnitine, coenzyme Q10 or taurine that exert antioxidant and anti-inflammatory activities [55] which may counteract some of the detrimental effects of other nutrients commonly found in meat like heme iron or saturated fatty acids and therefore also may benefit blood coagulation parameters. Therefore, the lack of association of meat intake with coagulation parameters in our study appears to be supported by the current scientific literature.

### 4.4. Fruits and Vegetables

In our study, for total vegetable and green leafy vegetable intake, no significant association with any blood coagulation parameter could be reported. However, for total fruit intake a positive association with aPTT was observed before adjusting for multiple testing. While there is emerging evidence for the beneficial effects of fruits and vegetable consumption on overall CVD risk [3,56], specific effects on coagulation parameters are not well established in the current literature. Possibly due to their antioxidant activity, the combined intake of fruits and vegetables has been associated with decreased fibrinogen values [57] and also, specific fruits like kiwis seem to have a fibrinogen lowering effect [58]. Therefore, positive effects on other markers of coagulation (not measured here) seem to be possible and could explain the observed anti-coagulant effect of fruit intake in our study, indicated by a positive association with aPTT. Fruits on average also appear to exert a more potent antioxidant activity, quantified by the ORAC (Oxygen Radical Absorbance Capacity) value, compared to vegetables [59,60] which could also explain the different results for those food items. Especially the lack of association of green leafy vegetables rich in vitamin K1 [61] could be due to their low intake amounts in our study with a mean value of only around 23.9 g per day.

### 4.5. Strengths and Limitations

Our study had several strengths such as a large number of participants, and the particular analysis of food groups instead of specific nutrients which were mainly investigated in previous studies. However, also some limitations exist. While our study measured nine different hemostatic parameters, there are of course other relevant markers of the coagulation system we did not analyze such as factor VII, the Von Willebrand factor or factor X which should be examined in further studies. The cross-sectional nature of our study does preclude any causal conclusions. Also, information about other comorbidities—besides hypertension, diabetes, high blood cholesterol (non-HDLc), or obesity (BMI)—or further specific blood markers such as sex hormones that could have been influenced by the intake of specific food items was not available in our dataset. Therefore, we could not adjust for additional diseases or conditions of the participants which could possibly have affected the measurements of hemostatic parameters. Further, the results may not be completely transferable to other populations or ethnic groups due to different environmental and congenital factors that could influence the metabolism of those individuals in a different manner. Finally, the self-reported dietary intake data assessed via 24-h food lists and a food frequency questionnaire are known to be unprecise and prone to recall bias.

## 5. Conclusions

While consumption of fruits and vegetables were not clearly associated with hemostatic markers, the results for animal-derived products are more mixed. For total fish, eggs and meat, no significant associations were observed whereas dairy product and butter consumption were associated with D-dimer and protein C concentrations. These findings need to be evaluated in independent prospective studies.

## Figures and Tables

**Table 1:** Overview of measured laboratory variables.

Laboratory Parameter	Method	Testing Device	Reference Value
Adjustment variables			
Total cholesterol (TC)	Enzymatic method	Cobas 8000 c702 Roche chemistry analyzer, Hoffman-La Roche AG Basel	<200 mg/dL
High-density lipoprotein cholesterol (HDLc)	Enzymatic method		>45 mg/dL
Triglycerides (TG)	Enzymatic method		<200 mg/dL
Gamma-glutamyl transferase (GGT)	IFCC method ^a^		Males: <60 U/L Females: <40 U/L
Hemostatic variables (dependent variables)			
Antithrombin III	Chromogenic activity assay	Innovance Antithrombin, SCS cleaner, Siemens Eschborn	83–118%
D-dimers	Particle-enhanced immunoturbidimetric assay	Innovance D-Dimer Kit, Siemens Eschborn	<500 μg/L
Factor VIII	Photometry	Coagulation factor VIII deficient plasma, Pathromtin SL, CaCl_2_, Siemens Eschborn	70–150%
Fibrinogen	Photometry and turbidimetry	Multifibern U, Siemens Eschborn	210–400 mg/dL
Protein C	Photometry	Berichrom Protein C, Siemens Healthcare	70–140%
Protein S	Photometry	Hemoclot Protein S, OVB buffer, CaCl_2_, SCS cleaner	Males: 73–130% Females: 52–126%
Activated partial thromboplastin time (aPTT)	Photometry	Pathromtin SL, CaCl_2_ solution, Actin FS, Siemens Eschborn	26–36 s
Quick value	Photometry	Thromborel S, Siemens Eschborn	82–125%
International thromboplastin time (INR)	Prothrombin ratio (calculated) ^b^		0.9–1.15
High-sensitivity C-reactive Protein (hs-CRP)	High-sensitivity latex-enhanced nephelometric assay	BN II System analyzer, Dade Behring	<3 mg/L ^c^

^a^ according to [28]; ^b^ according to [29]; ^c^ values below 1.00 mg/L were rounded down to 0.35 mg/L.

**Table 2:** Characteristics of the study participants.

	Total	Males	Females	
	n = 595	n = 263	n = 332	
Characteristics	Median (25th and 75th Percentiles)			*p*-Value
Age [years]	63	64	63	0.842
	(58; 68)	(58; 68)	(59; 68)	
BMI [kg/m^2^]	27.12	27.78	26.4	<0.001 *
	(23.98; 30.32)	(25.33; 30.67)	(23.3; 29.77)	
Waist circumference [cm]	93.1	99.4	86.05	<0.001 *
	(82.5; 102.35)	(91.75; 107.5)	(78; 96.32)	
Energy (kilocalories) [kcal/d]	1723.6	2049.9	1532.85	<0.001 *
	(1487.4; 2052.25)	(1809.9; 2298.1)	(1380.55; 1704.88)	
Alcohol consumption [g/d]	5.17	14.71	2.59	<0.001 *
	(2.19; 14.19)	(6.77; 25.94)	(1.59; 5.02)	
Non-HDL [mg/dL]	146.2	145	147.28	0.384
	(121; 171.84)	(115; 172.59)	(124.83; 170.56)	
FLI	51.88	79.85	34.28	<0.001 *
	(23.19; 88.33)	(42.88; 96.59)	(15.67; 72.6)	
hs-CRP [mg/L]	1 (1; 3)	1 (1; 2.5)	1 (1; 3)	0.25
	**n (%)**			
Education [years]				
≤12 years	359 (60.34%)	144 (54.75%)	215 (64.76%)	<0.001 *
>12 years	236 (39.66%)	119 (45.25%)	117 (35.24%)	
Physical activity				
≥2 h/week	229 (38.49%)	103 (39.16%)	126 (37.95%)	0.867
1 h/week	198 (33.28%)	84 (31.94%)	114 (34.34%)	
<1 h/week	71 (11.93%)	34 (12.93%)	37 (11.14%)	
(almost) no activity	97 (16.30%)	42 (15.97%)	55 (16.57%)	
Smoking				
Current smoker	72 (12.10%)	33 (12.55%)	39 (11.75%)	0.066
Former smoker	259 (43.53%)	127 (48.29%)	132 (39.76%)	
Never smoker	264 (44.37%)	103 (39.16%)	161 (48.49%)	
Hypertension				
Yes	272 (45.71%)	147 (55.89%)	125 (37.65%)	<0.001 *
No	323 (54.29%)	116 (44.11%)	207 (62.35%)	
Diabetes				
Yes	43 (7.23%)	20 (7.65%)	23 (6.93%)	0.753
No	552 (92.77%)	243 (92.4%)	309 (93.07%)	
BMI				
<18.5	3 (0.50%)	0 (0.00%)	3 (0.90%)	<0.001 *
18.5–24.9	186 (31.26%)	61 (23.19%)	125 (37.65%)	
25–29.9	246 (41.34%)	122 (46.39%)	124 (37.35%)	
>30	160 (26.89%)	80 (30.41%)	80 (24.10%)	

* *p* < 0.05.

**Table 3:** Plasma concentrations of blood coagulation factors in all participants and by sex.

	Total	Males	Females	
	n = 595	n = 263	n = 332	
Coagulation Factors	Median (25th and 75th Percentiles)			*p*-Value
Antithrombin III [mg/dL]	102.9	98.8	105.2	<0.001 *
	(96.15; 109)	(93.35; 105.55)	(99.1; 110.8)	
D-dimers [µg/L]	403	405	402	0.407
	(305; 544.5)	(315.5; 561)	(301.25; 532.25)	
Factor VIII [%]	120.3	118.4	123.5	0.13
	(96.6; 143.1)	(93.95; 140.2)	(99.92; 145.02)	
Fibrinogen [mg/dL]	293.5	285.7	299.15	0.054
	(261.05; 328.7)	(258.95; 322.1)	(262.92; 332.72)	
Protein C [%]	124.2	117.5	129.1	<0.001 *
	(111.45; 139.45)	(109.2; 131.7)	(116.12; 142.65)	
Protein S [%]	125.1	131.7	119.4	<0.001 *
	(105.6; 145.7)	(112.05; 157.7)	(101.3; 137.62)	
aPTT [s]	30.7	31.1	30.3	0.004
	(28.7; 32.9)	(29.4; 33.35)	(28.5; 32.7)	
Quick value [%]	108.7	106.7	110.45	<0.001 *
	(102.15; 114.9)	(100.25; 112.4)	(104.08; 115.82)	
INR	0.96	0.97	0.94	<0.001 *
	(0.92; 1)	(0.93; 1.01)	(0.91; 0.98)	

* p < 0.05.

**Table 4:** Habitual food consumption in all participants and by sex.

	Total	Males	Females	
	n = 595	n = 263	n = 332	
Food Groups [g/d]	Median (25th and 75th Percentiles)			*p*-Value
Total fruits	144.8	134.2	150.3	0.008
	(88.15; 213.55)	(75; 211.6)	(102.97; 216.5)	
Total vegetables	163.9	147.4	178.4	<0.001 *
	(134.5; 200.65)	(122.6; 178.05)	(148.7; 217.5)	
Green leafy vegetables	23.9	24.4	23.8	0.085
	(17.5; 32.35)	(18.15; 32.35)	(16.7; 32.2)	
Total meat	99	133.7	77.6	<0.001 *
	(73.5; 131.9)	(111.05; 160.05)	(64.57; 94.95)	
Total fish	18.2	19	17.35	0.001
	(12.35; 25.9)	(13.65; 27)	(11.67; 24.95)	
Total eggs	15.7	15.9	14.6	0.301
	(11.3; 22.2)	(11.6; 22.7)	(11.07; 21.63)	
Dairy products (w/o butter)	179.2 (120.85; 260)	154.4 (103.8; 228.9)	200.4 (135.2; 281.33)	<0.001 *
Cheese	27.8	29	26.85	0.062
	(19.3; 37.5)	(19.65; 41.4)	(19.17; 35.8)	
Butter	14.3	16.2	12.65	<0.001 *
	(8.95; 17.1)	(10.35; 21.5)	(7.68; 15.43)	

* *p* < 0.05.

**Table 5:** Association between habitual consumption of fruits, vegetables and green leafy vegetables [100 g/d] and blood coagulation parameters (dependent variables) ^a^.

	ß-Estimate	95% CI	*p*-Value	FDR Adjusted *p*-Value
Total fruit consumption [per 100 g/d]				
Antithrombin III [mg/dL]	0.125	−0.943; 1.192	0.819	1
Ln D-dimers [µg/L]	0.033	−0.022; 0.088	0.238	0.916
Ln Factor VIII [%]	−0.001	−0.034; 0.032	0.949	1
Ln Fibrinogen D [mg/dL]	−0.004	−0.023; 0.016	0.726	1
Protein C [%]	0.559	−1.305; 2.424	0.556	1
Ln Protein S [%]	−0.007	−0.034; 0.019	0.586	1
aPTT [s]	0.387	0.024; 0.75	0.036 ^b^	0.583
Quick value [%]	0.171	−0.832; 1.173	0.738	1
INR	−0.001	−0.007; 0.005	0.743	1
Total vegetable consumption [per 100 g/d]				
Antithrombin III [mg/dL]	−0.98	−2.676; 0.717	0.257	0.916
Ln D-dimers [µg/L]	0.023	−0.066; 0.113	0.609	1
Ln Factor VIII [%]	−0.005	−0.057; 0.047	0.856	1
Ln Fibrinogen D [mg/dL]	−0.012	−0.044; 0.02	0.47	1
Protein C [%]	−1.387	−4.357; 1.583	0.36	1
Ln Protein S [%]	−0.005	−0.048; 0.038	0.822	1
aPTT [s]	−0.302	−0.889; 0.284	0.312	0.972
Quick value [%]	0.03	−1.568; 1.628	0.97	1
INR	0	−0.01; 0.009	0.996	1
Green leafy vegetables consumption [per 100 g/d]				
Antithrombin III [mg/dL]	−2.192	−9.639; 5.254	0.563	1
Ln D-dimers [µg/L]	−0.045	−0.438; 0.349	0.823	1
Ln Factor VIII [%]	−0.131	−0.359; 0.097	0.26	0.916
Ln Fibrinogen D [mg/dL]	−0.09	−0.23; 0.05	0.209	0.916
Protein C [%]	−0.835	−13.861; 12.191	0.9	1
Ln Protein S [%]	−0.164	−0.352; 0.025	0.089	0.714
aPTT [s]	−0.705	−3.299; 1.888	0.594	1
Quick value [%]	0.826	−6.255; 7.906	0.819	1
INR	−0.005	−0.047; 0.037	0.807	1

^a^ linear regression models adjusted for sex, age, physical activity, education years, smoking status, diabetes, hypertension, calorie intake, alcohol consumption, non-HDL cholesterol and BMI. CI, confidence interval; FDR, false discovery rate; ^b^ *p* < 0.05.

**Table 6:** Association between habitual consumption of foods of animal origin [100 g/d] and blood coagulation parameters (dependent variables) ^a^.

	ß-Estimate	95% CI	*p*-Value	FDR Adjusted *p*-Value
Total meat consumption [per 100 g/d]				
Antithrombin III [mg/dL]	2.838	−0.466; 6.143	0.092	0.714
Ln D-dimers [µg/L]	−0.074	−0.25; 0.102	0.41	1
Ln Factor VIII [%]	0.014	−0.086; 0.114	0.785	1
Ln Fibrinogen D [mg/dL]	0.014	−0.048; 0.077	0.65	1
Protein C [%]	1.745	−4.047; 7.537	0.554	1
Ln Protein S [%]	−0.024	−0.109; 0.06	0.568	1
aPTT [s]	0.075	−1.067; 1.218	0.897	1
Quick value [%]	−1.943	−5.089; 1.203	0.226	0.916
INR	0.011	−0.007; 0.03	0.235	0.916
Total fish consumption [per 100 g/d]				
Antithrombin III [mg/dL]	−3.373	−8.922; 2.176	0.233	0.916
Ln D-dimers [µg/L]	−0.038	−0.337; 0.26	0.8	1
Ln Factor VIII [%]	−0.011	−0.18; 0.157	0.895	1
Ln Fibrinogen D [mg/dL]	−0.061	−0.166; 0.044	0.253	0.916
Protein C [%]	−0.124	−9.845; 9.598	0.98	1
Ln Protein S [%]	−0.047	−0.188; 0.094	0.514	1
aPTT [s]	−0.251	−2.187; 1.685	0.799	1
Quick value [%]	−0.041	−5.328; 5.246	0.988	1
INR	0	−0.031; 0.032	0.976	1
Total egg consumption [per 100 g/d]				
Antithrombin III [mg/dL]	5.191	−1.551; 11.933	0.131	0.884
Ln D-dimers [µg/L]	0.018	−0.339; 0.375	0.92	1
Ln Factor VIII [%]	0.039	−0.168; 0.246	0.711	1
Ln Fibrinogen D [mg/dL]	−0.022	−0.151; 0.107	0.739	1
Protein C [%]	3.827	−7.978; 15.633	0.525	1
Ln Protein S [%]	−0.002	−0.173; 0.169	0.985	1
aPTT [s]	−1.265	−3.624; 1.094	0.293	0.972
Quick value [%]	1.142	−5.261; 7.544	0.726	1
INR	−0.007	−0.045; 0.032	0.734	1

^a^ linear regression models adjusted for sex, age, physical activity, education years, smoking status, diabetes, hypertension, calorie intake, alcohol consumption, non-HDL cholesterol and BMI. CI, confidence interval; FDR, false discovery rate.

**Table 7:** Association between habitual consumption of dairy products (w/o butter), cheese and butter [100 g/d] and blood coagulation parameters (dependent variables) ^a^.

	ß-Estimate	95% CI	*p*-Value	FDR Adjusted *p*-Value
Dairy products (w/o butter) consumption [per 100 g/d]				
Antithrombin III [mg/dL]	−1.132	−1.99; −0.275	0.01 ^b^	0.27
Ln D-dimers [µg/L]	0.049	0.004; 0.094	0.032 ^b^	0.583
Ln Factor VIII [%]	0.003	−0.023; 0.029	0.808	1
Ln Fibrinogen D [mg/dL]	0.002	−0.014; 0.018	0.782	1
Protein C [%]	−2.644	−4.137; −1.152	0.001 ^b^	0.04 ^b^
Ln Protein S [%]	0.015	−0.007; 0.036	0.19	0.916
aPTT [s]	0.029	−0.265; 0.324	0.845	1
Quick value [%]	−0.083	−0.896; 0.73	0.841	1
INR	0.001	−0.004; 0.005	0.791	1
Cheese consumption [per 100 g/d]				
Antithrombin III [mg/dL]	1.359	−4.932; 7.649	0.672	1
Ln D-dimers [µg/L]	0.173	−0.16; 0.507	0.308	0.972
Ln Factor VIII [%]	0.069	−0.122; 0.26	0.478	1
Ln Fibrinogen D [mg/dL]	−0.016	−0.135; 0.103	0.786	1
Protein C [%]	2.31	−8.705; 13.324	0.681	1
Ln Protein S [%]	−0.115	−0.274; 0.045	0.159	0.916
aPTT [s]	0.438	−1.758; 2.633	0.695	1
Quick value [%]	0.001	−5.913; 5.915	1	1
INR	−0.001	−0.037; 0.034	0.94	1
Butter consumption [per 100 g/d]				
Antithrombin III [mg/dL]	8.939	−5.68; 23.558	0.23	0.916
Ln D-dimers [µg/L]	1.429	0.669; 2.19	<0.001 ^b^	<0.001 ^b^
Ln Factor VIII [%]	0.145	−0.304; 0.595	0.526	1
Ln Fibrinogen D [mg/dL]	0.231	−0.042; 0.504	0.097	0.714
Protein C [%]	25.909	0.367; 51.451	0.047 ^b^	0.59
Ln Protein S [%]	−0.111	−0.483; 0.261	0.559	1
aPTT [s]	−1.503	−6.504; 3.498	0.555	1
Quick value [%]	13.207	−0.529; 26.943	0.059	0.597
INR	−0.082	−0.163; 0	0.051	0.59

^a^ linear regression models adjusted for sex, age, physical activity, education years, smoking status, diabetes, hypertension, calorie intake, alcohol consumption, non-HDL cholesterol and BMI. CI, confidence interval; FDR, false discovery rate; ^b^ *p* < 0.05.

## Data Availability

The data are subject to national data protection laws, and restrictions were imposed by the Ethics Committee of the Bavarian Chamber of Physicians to ensure data privacy of the study participants. Therefore, data cannot be made freely available in a public repository. However, data can be requested through an individual project agreement with KORA via the online portal KORA (https://www.helmholtz-munich.de/en/epi accessed on 6 December 2023).

## Supplementary Materials

The following supporting information can be downloaded at https://www.mdpi.com/article/10.3390/nu16030432/s1: Table S1: Overview of participants with values outside the reference range for hemostatic parameters. Table S2: Association between habitual consumption of fruits, vegetables and green leafy vegetables and blood coagulation parameters additionally adjusted for FLI. Table S3: Association between habitual consumption of foods of animal origin and blood coagulation parameters additionally adjusted for FLI. Table S4: Association between habitual consumption of dairy products (w/o butter), cheese and butter and blood coagulation parameters additionally adjusted for FLI.
