# Supplementary material for: Is the Habitual Dietary Intake of Foods of Plant or Animal Origin Associated with Circulating Hemostatic Factors?—Results of the Population-Based KORA-Fit Study
Source: Nutrients. 2024 Jan 31;16(3):432. doi: 10.3390/nu16030432 (PMC10857183; doi:10.3390/nu16030432)
Supplement: Supplementary file 1 [file nutrients-16-00432-s001.zip › nutrients-2834545-supplementary.pdf]

Supplementary data

Table S1.
Overview of participants with values below or above the reference range for hemostatic parameters

	**Reference Range**	**n (%) below the reference range**	**n (%) above the reference range**
Antithrombin III	83 - 118%	14 (2.35%)	42 (7.06%)
D-dimers	<500 µg/l		190 (31.93%)
Factor VIII	70 - 150%	24 (4.03%)	114 (19.16%)
Fibrinogen	210 – 400 mg/dl	17 (2.86%)	27 (4.54%)
Protein C	70 - 140%	4 (0.67%)	145 (24.37%)
Protein S	Males: 73 - 130%<br>Females: 52 -	Males: 4 (1.52%)<br>Females: 0	Males: 137 (52.09%)<br>Females: 138
Activated partial thromboplastin time (aPTT)	26 - 36 s	23 (3.87%)	39 (6.56%)
Quick value	82 - 125%	4 (0.67%)	15 (2.52%)
International thromboplastin time (INR)	0.9 - 1.15	61 (10.25%)	3 (0.50%)

Linear regression models adjusted for Fatty Liver Index (FLI), replacing BMI

Table S2.
Association between habitual consumption of fruits, vegetables and green leafy vegetables [100g/d] and blood coagulation parameters (dependent variables), additionally adjusted for FLI [a]

	**ß-estimate**	**95% CI**	**p-value**	**FDR adjusted p-value**
Total fruit consumption [100g/d]				
Antithrombin III [mg/dl]	-0.022	-1.1; 1.057	0.969	0.995
Ln D-dimers [µg/l]	0.038	-0.017; 0.093	0.176	0.881
Ln Factor VIII [%]	0	-0.032; 0.033	0.983	0.995
Ln Fibrinogen D [mg/dl]	-0.002	-0.022; 0.018	0.875	0.995
Protein C [%]	0.571	-1.286; 2.427	0.546	0.995
Ln Protein S [%]	-0.006	-0.033; 0.021	0.687	0.995
aPTT [s]	0.384	0.022; 0.746	0.038[b]	0.58
Quick value [%]	0.216	-0.779; 1.211	0.67	0.995
INR	-0.001	-0.007; 0.005	0.67	0.995
Total vegetable consumption [100g/d]				
Antithrombin III [mg/dl]	-1.085	-2.801; 0.631	0.215	0.917
Ln D-dimers [µg/l]	0.027	-0.063; 0.118	0.552	0.995
Ln Factor VIII [%]	-0.005	-0.057; 0.046	0.845	0.995
Ln Fibrinogen D [mg/dl]	-0.01	-0.042; 0.022	0.546	0.995
Protein C [%]	-1.392	-4.352; 1.569	0.356	0.995
Ln Protein S [%]	-0.003	-0.046; 0.04	0.887	0.995
aPTT [s]	-0.314	-0.9; 0.271	0.292	0.985
Quick value [%]	0.103	-1.486; 1.692	0.899	0.995
INR	0	-0.01; 0.009	0.923	0.995
Green leafy vegetables consumption [100g/d]				
Antithrombin III [mg/dl]	-2.457	-9.986; 5.071	0.522	0.995
Ln D-dimers [µg/l]	-0.032	-0.429; 0.364	0.872	0.995
Ln Factor VIII [%]	-0.144	-0.371; 0.083	0.212	0.917
Ln Fibrinogen D [mg/dl]	-0.085	-0.227; 0.056	0.236	0.956
Protein C [%]	-1.415	-14.405; 11.574	0.831	0.995
Ln Protein S [%]	-0.163	-0.351; 0.026	0.091	0.737
aPTT [s]	-0.651	-3.24; 1.937	0.621	0.995
Quick value [%]	0.666	-6.367; 7.699	0.852	0.995
INR	-0.004	-0.046; 0.038	0.839	0.995

[a]linear regression models adjusted for sex, age, physical activity, education years, smoking status, diabetes, hypertension, calorie intake, alcohol consumption, non-HDL cholesterol and FLI. CI, confidence interval; FDR false discovery rate; [b]$p<0.05$

Table S3.
Association between habitual consumption of foods of animal origin [100g/d] and blood coagulation parameters (dependent variables), additionally adjusted for FLI [a]

	**ß-estimate**	**95% CI**	**p-value**	**FDR adjusted p-value**
	Total meat consumption [100g/d]			
Antithrombin III [mg/dl]	1.396	-1.87; 4.662	0.401	0.995
Ln D-dimers [µg/l]	-0.016	-0.19; 0.157	0.854	0.995
Ln Factor VIII [%]	0.002	-0.096; 0.099	0.973	0.995
Ln Fibrinogen D [mg/dl]	0.042	-0.019; 0.104	0.178	0.881
Protein C [%]	0.798	-4.824; 6.421	0.78	0.995
Ln Protein S [%]	-0.014	-0.096; 0.068	0.744	0.995
aPTT [s]	0.298	-0.815; 1.411	0.599	0.995
Quick value [%]	-2.381	-5.429; 0.666	0.125	0.881
INR	0.014	-0.004; 0.032	0.136	0.881
	Total fish consumption [100g/d]			
Antithrombin III [mg/dl]	-3.867	-9.467; 1.734	0.176	0.881
Ln D-dimers [µg/l]	-0.018	-0.318; 0.282	0.908	0.995
Ln Factor VIII [%]	-0.011	-0.178; 0.156	0.897	0.995
Ln Fibrinogen D [mg/dl]	-0.046	-0.152; 0.059	0.39	0.995
Protein C [%]	-0.217	-9.88; 9.446	0.965	0.995
Ln Protein S [%]	-0.043	-0.184; 0.098	0.55	0.995
aPTT [s]	-0.169	-2.099; 1.761	0.863	0.995
Quick value [%]	-0.228	-5.473; 5.017	0.932	0.995
INR	0.002	-0.03; 0.033	0.924	0.995
	Total egg consumption [100g/d]			
Antithrombin III [mg/dl]	3.768	-3.014; 10.55	0.276	0.985
Ln D-dimers [µg/l]	0.08	-0.277; 0.438	0.66	0.995
Ln Factor VIII [%]	0.032	-0.173; 0.236	0.762	0.995
Ln Fibrinogen D [mg/dl]	0.009	-0.121; 0.138	0.897	0.995
Protein C [%]	3.055	-8.622; 14.731	0.608	0.995
Ln Protein S [%]	0.007	-0.163; 0.177	0.934	0.995
aPTT [s]	-1.062	-3.403; 1.279	0.373	0.995
Quick value [%]	0.537	-5.801; 6.875	0.868	0.995
INR	-0.004	-0.042; 0.034	0.841	0.995

[a]linear regression models adjusted for sex, age, physical activity, education years, smoking status, diabetes, hypertension, calorie intake, alcohol consumption, non-HDL cholesterol and FLI. CI, confidence interval; FDR false discovery rate

Table S4.
Association between habitual consumption of dairy products (w/o butter), cheese and butter [100g/d] and blood coagulation parameters (dependent variables), additionally adjusted for FLI [a]

	**ß-estimate**	**95% CI**	**p-value**	**FDR adjusted p-value**
Dairy products (w/o butter) consumption [100g/d]				
Antithrombin III [mg/dl]	-1.23	-2.095; -0.365	0.005[b]	0.135
Ln D-dimers [µg/l]	0.053	0.008; 0.098	0.022[b]	0.445
Ln Factor VIII [%]	0.004	-0.022; 0.03	0.75	0.995
Ln Fibrinogen D [mg/dl]	0.004	-0.012; 0.021	0.599	0.995
Protein C [%]	-2.615	-4.1; -1.13	0.001[b]	0.081
Ln Protein S [%]	0.016	-0.006; 0.037	0.158	0.881
aPTT [s]	0.03	-0.263; 0.324	0.839	0.995
Quick value [%]	-0.066	-0.873; 0.741	0.872	0.995
INR	0.001	-0.004; 0.005	0.826	0.995
Cheese consumption [100g/d]				
Antithrombin III [mg/dl]	0.714	-5.639; 7.066	0.825	0.995
Ln D-dimers [µg/l]	0.195	-0.14; 0.53	0.254	0.98
Ln Factor VIII [%]	0.073	-0.117; 0.262	0.45	0.995
Ln Fibrinogen D [mg/dl]	-0.001	-0.121; 0.119	0.983	0.995
Protein C [%]	2.253	-8.702; 13.208	0.686	0.995
Ln Protein S [%]	-0.108	-0.267; 0.052	0.185	0.881
aPTT [s]	0.497	-1.691; 2.684	0.656	0.995
Quick value [%]	-0.01	-5.891; 5.872	0.997	0.997
INR	-0.001	-0.036; 0.034	0.959	0.995
Butter consumption [100g/d]				
Antithrombin III [mg/dl]	13.739	-0.804; 28.281	0.064	0.63
Ln D-dimers [µg/l]	1.198	0.442; 1.953	0.002[b]	0.081
Ln Factor VIII [%]	0.126	-0.315; 0.567	0.574	0.995
Ln Fibrinogen D [mg/dl]	0.147	-0.125; 0.42	0.289	0.985
Protein C [%]	25.914	0.799; 51.029	0.043[b]	0.58
Ln Protein S [%]	-0.162	-0.529; 0.204	0.385	0.995
aPTT [s]	-1.694	-6.61; 3.223	0.499	0.995
Quick value [%]	12.438	-1.006; 25.881	0.07	0.63
INR	-0.076	-0.156; 0.004	0.062	0.63

[a]linear regression models adjusted for sex, age, physical activity, education years, smoking status, diabetes, hypertension, calorie intake, alcohol consumption, non-HDL cholesterol and FLI. CI, confidence interval; FDR false discovery rate; [b]$p<0.05$
